# Supplementary material for: Progesterone influences cytoplasmic maturation in porcine oocytes developing in vitro
Source: PeerJ. 2016 Sep 15;4:e2454. doi: 10.7717/peerj.2454 (PMC5028735; doi:10.7717/peerj.2454)
Supplement: Data S1 [file peerj-04-2454-s001.pdf]

| | | | | | | 1st | | | 2nd | | | 3rd | | |
|---|---|---|---|---|---|---|---|---|---|---|---|---|---|---|
| Group | concentrations | total COCs | mean of MII rate | SD | P value | COCs | MII | MII rate | COCs | MII | MII rate | COCs | MII | MII rate |
| Normal IVM medium add P4 | 0 | 136 | 0.7630 | 0.0519 | 0.705 | 41 | 32 | 0.7805 | 44 | 31 | 0.7045 | 51 | 41 | 0.8039 |
| | 10 μM | 146 | 0.7979 | 0.0441 | | 40 | 30 | 0.7500 | 49 | 41 | 0.8367 | 57 | 46 | 0.8070 |
| | 100 μM | 158 | 0.7751 | 0.0546 | | 43 | 31 | 0.7209 | 53 | 44 | 0.8302 | 62 | 48 | 0.7742 |
| IVM medium(No FSH, LH, PFF) add P4 | 0 | 250 | 0.4940 | 0.0290 | 0.386 | 74 | 39 | 0.5270 | 85 | 41 | 0.4824 | 91 | 43 | 0.4725 |
| | 10 μM | 254 | 0.5175 | 0.0256 | | 76 | 41 | 0.5395 | 84 | 44 | 0.5238 | 94 | 46 | 0.4894 |
| | 100 μM | 248 | 0.5261 | 0.0271 | | 74 | 41 | 0.5541 | 82 | 43 | 0.5244 | 92 | 46 | 0.5000 |
| Normal IVM medium add RU486 | 0 | 207 | 0.7525 | 0.0415 | 0.000 | 59 | 47 | 0.7966 | 71 | 53 | 0.7465 | 77 | 55 | 0.7143 |
| | 10 μM | 215 | 0.4956 | 0.0278 | | 61 | 32 | 0.5246 | 73 | 36 | 0.4932 | 81 | 38 | 0.4691 |
| | 25 μM | 208 | 0.4832 | 0.0294 | | 60 | 31 | 0.5167 | 70 | 33 | 0.4714 | 78 | 36 | 0.4615 |
| | 25 μM+100 μMP4 | 139 | 0.4900 | 0.0388 | | 45 | 24 | 0.5333 | 48 | 22 | 0.4583 | 46 | 22 | 0.4783 |
