# Supplementary material for: Progesterone influences cytoplasmic maturation in porcine oocytes developing in vitro
Source: PeerJ. 2016 Sep 15;4:e2454. doi: 10.7717/peerj.2454 (PMC5028735; doi:10.7717/peerj.2454)
Supplement: Data S2 [file peerj-04-2454-s002.pdf]

Raw data of ROS

		mean	sd	P value	letter
1	Control	9.12	3.00	1-2, P=0.006	a
2	100 uM P4	4.80	1.57	1-3, P<0.01	b
3	25 uM RU486	16.54	4.75	2-3, P<0.01	c

Raw data of GSH

		mean	sd	P value	letter
1	Control	62.84	7.27	1-2, P=0.032	a
2	100 uM P4	68.14	4.81	1-3, P<0.01	b
3	25 uM RU486	45.82	8.33	2-3, P<0.01	c
