# Supplementary material for: Progesterone influences cytoplasmic maturation in porcine oocytes developing in vitro
Source: PeerJ. 2016 Sep 15;4:e2454. doi: 10.7717/peerj.2454 (PMC5028735; doi:10.7717/peerj.2454)
Supplement: Data S3 [file peerj-04-2454-s003.pdf]

Real-time RT-PCR analysis of the expression patterns of maternal mRNAs at MII stage

cdc2

		mean	SD	p value	letter
1	Control	1.000	0.044	1-2, P=0.002	a
2	100 μM P4	1.443	0.153	1-3, P=0.002	b
3	25 μM RU486	0.550	0.096	2-3, P<0.001	c

Cyclin B1

		mean	SD	p value	letter
1	Control	1.000	0.035	1-2, P=0.021	a
2	100 μM P4	1.230	0.104	1-3, P=0.198	b
3	25 μM RU486	0.893	0.112	2-3, P=0.004	a

MPF activity in MII oocytes

		mean	SD	p value	letter
1	Control	0.293	0.015	1-2, P=0.128	a
2	100 μM P4	0.323	0.023	1-3, P=0.001	a
3	25 μM RU486	0.175	0.012	2-3, P<0.001	b
