# Supplementary material for: Progesterone influences cytoplasmic maturation in porcine oocytes developing in vitro
Source: PeerJ. 2016 Sep 15;4:e2454. doi: 10.7717/peerj.2454 (PMC5028735; doi:10.7717/peerj.2454)
Supplement: Data S4 [file peerj-04-2454-s004.pdf]

						1st			2nd			3rd		
Group	concentrations	total MII oocytes	mean of BL rate	SD	P value	MII	BL	BL rate	MII	BL	BL rate	MII	BL	BL rate
Normal IVM medium add P4	0	93	0.5304	0.0282	0.359	28	15	0.5357	27	15	0.5556	38	19	0.5000
	10 μ*M*	109	0.5126	0.0363		27	14	0.5185	38	18	0.4737	44	24	0.5455
	100 μ*M*	110	0.5534	0.0314		28	15	0.5357	39	23	0.5897	43	23	0.5349
IVM medium(No FSH,LH,PFF) add P4	0	114	0.3940	0.0187	0.003	36	14	0.3889	37	14	0.3784	41	17	0.4146
	10 μ*M*	106	0.4169	0.0256		31	13	0.4194	34	15	0.4412	41	16	0.3902
	100 μ*M*	125	0.5028	0.0265		39	19	0.4872	41	20	0.4878	45	24	0.5333
Normal IVM medium add RU486	0	144	0.4858	0.0309	0.002	44	21	0.4773	50	26	0.5200	50	23	0.4600
	10 μ*M*	93	0.3424	0.0257		27	9	0.3333	31	10	0.3226	35	13	0.3714
	25 μ*M*	90	0.3231	0.0427		28	9	0.3214	30	11	0.3667	32	9	0.2813
	25 μM+100 μMP4	65	0.3365	0.0483		23	9	0.3913	22	7	0.3182	20	6	0.3000
