# Supplementary material for: Progesterone influences cytoplasmic maturation in porcine oocytes developing in vitro
Source: PeerJ. 2016 Sep 15;4:e2454. doi: 10.7717/peerj.2454 (PMC5028735; doi:10.7717/peerj.2454)
Supplement: Data S5 [file peerj-04-2454-s005.pdf]

	Control	Apoptotic cell rate	P4 100uM	Apoptotic cell rate	RU486 25uM	Apoptotic cell rate
mean	41.95	0.033	47.04	0.024	40.28	0.080
SD	4.05	0.026	4.90	0.020	3.48	0.043
P value	1-2, p<0.0	1-2,p=0.036	1-3, p=0.233	1-3,p<0.01	2-3,p<0.01	2-3,p<0.01
letter	a		b		a	
blastocyst numbers	20		25		18	
1	42	2	41	2	44	7
2	44	2	42	1	42	6
3	45	3	54	1	42	7
4	47	1	53	2	36	2
5	38	3	41	0	48	1
6	43	1	40	0	43	2
7	44	0	43	0	37	4
8	44	1	38	1	42	5
9	35	0	39	2	45	3
10	37	0	44	1	40	2
11	42	2	54	0	38	4
12	44	1	48	0	36	4
13	43	2	53	0	38	3
14	45	0	45	0	37	1
15	37	2	50	2	37	1
16	42	1	49	1	42	1
17	45	3	48	2	37	3
18	47	0	53	2	41	4
19	32	2	48	1		
20	43	1	49	2		
21			50	2		
22			51	4		
23			48	1		
24			49	1		
25			46	1		
