# Supplementary material for: Progesterone influences cytoplasmic maturation in porcine oocytes developing in vitro
Source: PeerJ. 2016 Sep 15;4:e2454. doi: 10.7717/peerj.2454 (PMC5028735; doi:10.7717/peerj.2454)
Supplement: Data S6 [file peerj-04-2454-s006.pdf]

Relative mRNA expression levels of Bcl-2, Bax, and Casp3

Bcl-2

		mean	SD	p value	letter
1	Control	1.000	0.087	1-2,P=0.242	a
2	100 μM P4	1.090	0.070	1-3,P=0.003	a
3	25 μM RU486	0.670	0.095	2-3,P=0.001	b

Bax

		mean	SD	p value	letter
1	Control	1.000	0.104	1-2,P=0.271	a
2	100 μM P4	0.910	0.087	1-3,P<0.001	a
3	25 μM RU486	1.520	0.079	2-3,P<0.001	b

Casp3

		mean	SD	p value	letter
1	Control	1.000	0.096	1-2,P<0.001	a
2	100 μM P4	0.450	0.034	1-3,P<0.001	b
3	25 μM RU486	2.250	0.104	2-3,P<0.001	c
